# Comparative Proteomic Analysis of *Lactobacillus plantarum* WCFS1 and Δ*ctsR* Mutant Strains Under Physiological and Heat Stress Conditions

**DOI:** 10.3390/ijms130910680

**Published:** 2012-08-24

**Authors:** Pasquale Russo, María de la Luz Mohedano, Vittorio Capozzi, Pilar Fernández de Palencia, Paloma López, Giuseppe Spano, Daniela Fiocco

**Affiliations:** 1 Department of Agriculture, Food and Environment Sciences, Via Napoli 25, Foggia 71122, Italy; E-Mails: p.russo@unifg.it (P.R.); vittorio.capozzi@gmail.com (V.C.); 2 Biology Research Center, Department of Molecular Microbiology and Infection Biology, calle Ramiro de Maetzu 9, Madrid 28040, Spain; E-Mails: mmoheda@cib.csic.es (M.L.M.); pfpalencia@cib.csic.es (P.F.P.); plg@cib.csic.es (P.L.); 3 Department of Clinical and Experimental Medicine, University of Foggia, Via Pinto, 1, Foggia 71122, Italy; E-Mail: d.fiocco@unifg.it

**Keywords:** ClpE, CtsR, heat, *Lactobacillus plantarum*, stress

## Abstract

Among Gram-positive bacteria, CtsR (Class Three Stress gene Repressor) mainly regulates the expression of genes encoding the Clp ATPases and the ClpP protease. To gain a better understanding of the biological significance of the CtsR regulon in response to heat-shock conditions, we performed a global proteomic analysis of *Lactobacillus plantarum* WCFS1 and Δ*ctsR* mutant strains under optimal or heat stress temperatures. Total protein extracts from bacterial cells were analyzed by two-dimensional gel fractionation. By comparing maps from different culture conditions and different *L. plantarum* strains, image analysis revealed 23 spots with altered levels of expression. The proteomic analysis of *L. plantarum* WCFS1 and *ctsR* mutant strains confirms at the translational level the CtsR-mediated regulation of some members of the Clp family, as well as the heat induction of typical stress response genes. Heat activation of the putative CtsR regulon genes at transcriptional and translational levels, in the Δ*ctsR* mutant, suggests additional regulative mechanisms, as is the case of *hsp1*. Furthermore, isoforms of ClpE with different molecular mass were found, which might contribute to CtsR quality control. Our results could add new outlooks in order to determine the complex biological role of CtsR-mediated stress response in lactic acid bacteria.

## 1. Introduction

Microorganisms respond to stress by multiple adaptive mechanisms. Environmental signals can induce dramatic changes in the expression pattern of a variety of stress-related genes, encoding proteins that improve adaptation to the changing conditions. Heat is a source of stress to all living microorganisms. Increased temperature causes protein denaturation and subsequent aggregation, destabilizes macromolecules and alters membrane fluidity [1]. Temperature upshift induces expression of genes coding for heat shock proteins (HSPs). Some of these, such as GroEL, DnaK, small heat-shock proteins, and Clp ATPases, are molecular chaperones that assist re-folding of damaged cellular proteins [1]; others, such as ClpP and FtsH, act as proteases and degrade irreversibly damaged proteins [2].

The expression of eubacterial stress genes is primarily controlled at the transcriptional level, both positively and negatively. In *Bacillus subtilis*, which is considered a model organism of Gram-positive bacteria, heat shock genes have been classified, based on their transcriptional control mechanism [3]. The so-called “class III stress response genes” are negatively regulated by CtsR, a highly conserved transcriptional repressor originally identified in *B. subtilis* and subsequently found in most low GC% Gram-positive bacteria, where it mainly controls the expression of *clp* genes [4,5]. As a dimer, CtsR recognizes and binds a conserved hepta-nucleotide direct repeat (5′-RGTCADN NAN RGTCADN-3′) overlapping the promoter or its downstream region of regulated genes, thereby suppressing their expression [5,6]. CtsR DNA-binding activity is, itself, temperature-dependent [7]. In *B. subtilis*, heat stress inactivated CtsR is thereby specifically targeted to degradation by the adaptor protein McsB and its partner McsA; conversely in those low GC Gram-positive bacteria lacking McsB and McsA, their regulatory role seems to be driven by the ClpE chaperone [8].

*Lactobacillus plantarum* is a widespread lactic acid bacterium (LAB) species. It is present in several fermented food products of animal or plant origin, where it is either added as a starter culture, or is present as part of the indigenous microbiota [9]. *L. plantarum* is also a commensal microorganism of the human gut microbiota [10] and some strains possess probiotic properties [11,12]. The wide distribution of *L. plantarum* indicates its ability to adapt to different ecosystems, and studies of its stress response could shed light on the mechanisms underlying its high adaptability.

In *L. plantarum* WCFS1, CtsR regulates the transcription of the *ctsR-clpC* operon, *hsp1* and *ftsH* [13,14]. Transcriptional and genomic sequence analysis indicated that the CtsR regulon includes *clpP*, *clpB*, *clpE* and possibly other, apparently heat stress-unrelated genes. Consistent with the potentially large size of its regulon, *ctsR* deletion generated heat sensitivity and alteration of surface morphology in heat-stressed cells, thus suggesting that in *L. plantarum*, CtsR might play a major role in heat stress tolerance connected to its key role in controlling processes involved in protein quality [13,14]. Reference proteomic maps of *L. plantarum* have been determined [15,16] and several authors have recently studied proteome changes in heat-adapted [17], bile-treated [18] or alkaline-stressed [19] cells.

In this study, in order to confirm and/or identify additional roles of CtsR in *L. plantarum* WCFS1, the influence of Δ*ctsR* mutation under physiological and heat shock stress conditions was analyzed at the proteomic level.

## 2. Results and Discussion

### 2.1. Detection and Identification of Spots with Altered Expression Levels

**Figure 1:**
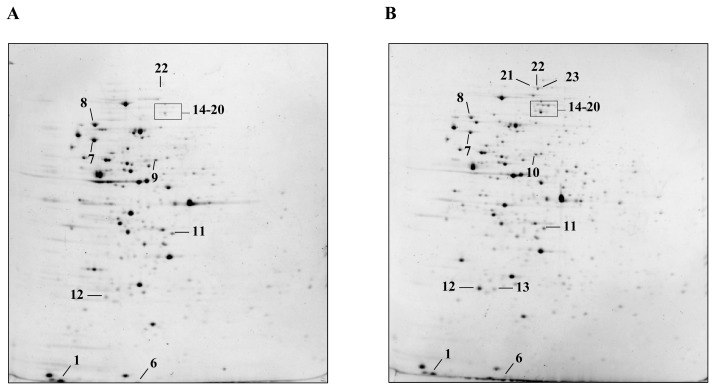

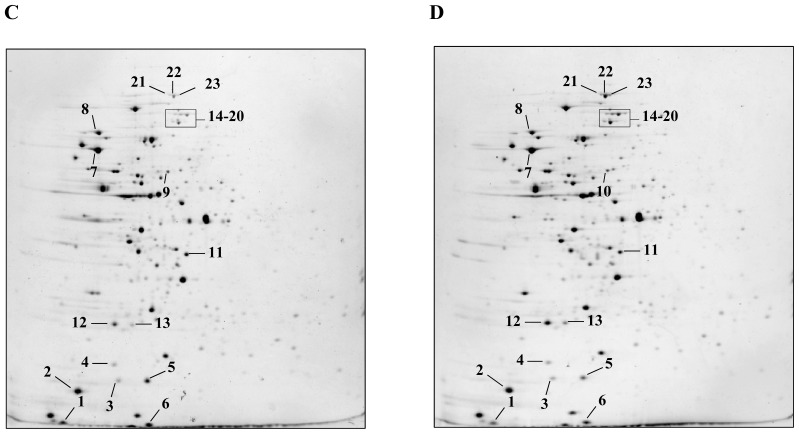
2-DE analysis of *L. plantarum* protein extracts. 2D-gel analysis of total protein extracts of *L. plantarum* WCFS1 wild type and Δ*ctsR* mutant strains, from either unstressed cultures (**A** and **B**, respectively) or after exposure to 42 °C for 30 min (**C** and **D**, respectively). Peptides were separated by IEF in the pI range of 4.0 to 7.0 in the first dimension and by 12% SDS-PAGE in the second dimension. Resolved peptides were visualized by SYPRO Ruby staining. Differentially expressed spots are indicated.

Total protein extracts from *L. plantarum* WCFS1 wild type and Δ*ctsR* mutant strains from unstressed or heat-treated cultures were analyzed by 2D-gel fractionation, using a linear pH gradient (pH 4.0–7.0) during electrofocusing. The second dimension exhibited a separation range from 10 to 250 kDa. Representative gels of the four experimental conditions are depicted in Figure 1.

Image analysis revealed a total of 305 ± 3 and 328 ± 4 well-resolved peptide spots for the wild type and the mutant grown at 30 °C, as well as 311 ± 10 and 333 ± 7 for the same strains after temperature upshift to 42 °C.

Quantification of the spot intensity, performed by PDQuest software analysis, pointed out statistically significant changes in the levels of 23 spots (Figure 1 and Table 1).

**Figure 2:**
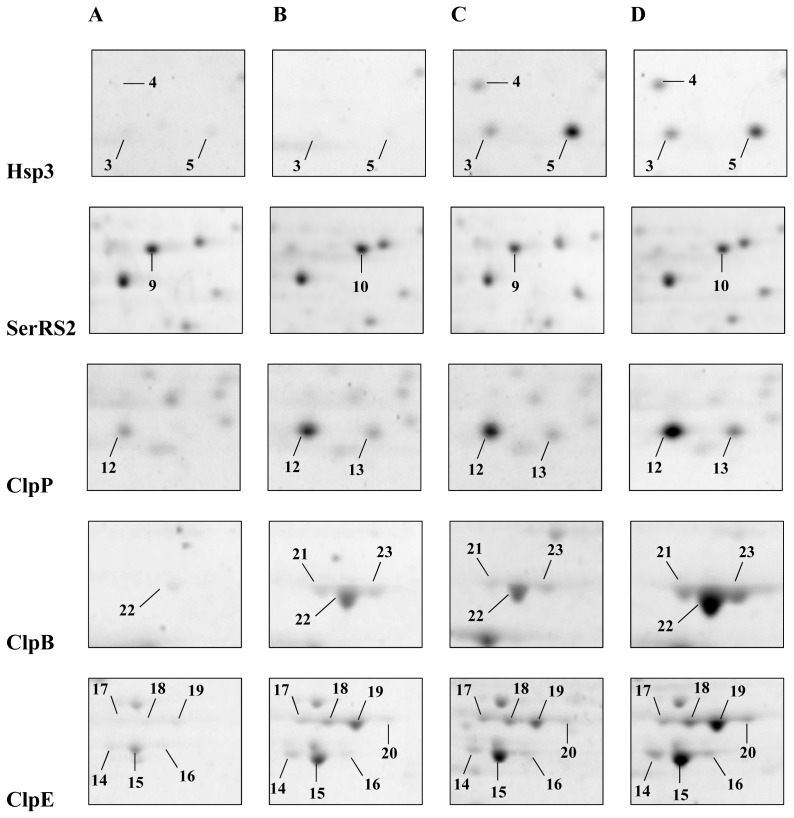
Focus on some spots of major interest. Spot images of the Hsp3, SerRS2, ClpP, ClpB, and ClpE isoforms in *L. plantarum* WCFS1 wild type (**A** and **C**) and Δ*ctsR* mutant (**B** and **D**) unstressed (**A** and **B**) or heat-treated (**C** and **D**) cultures.

Their identification, after *in situ* digestion with trypsin, by MALDI-TOF-TOF peptide mass fingerprinting, revealed that in several cases, different spots corresponded to essentially the same polypeptide and thus constituted different isoforms, presumably arising from *in vivo* post-translational modifications or due to artifacts occurring during sample handling. The characteristics of the investigated peptides are summarized in Table 1 and detailed maps of selected spots are reported in Figure 2.

In summary, the 23 analyzed spots corresponded to 10 proteins: the cold shock protein CspC (spot 1); the heat shock proteins Hsp1 (spot 2) and Hsp3 (spots 3–5); typical chaperonines GroES, GroEl and DnaK (spots 6, 7 and 8), the seryl-tRNA synthetase SerRS2 (spots 9–10) and three proteins ClpP, ClpE, ClpB belonging to Clp family (spots 11–23).

### 2.2. Influence of Heat Shock on Protein Levels

The heat shock response in both strains resulted in an increase in intensity of seven spots (2–8 in Figure 1) corresponding to five different peptides. In addition, spot quantification always indicated a greater heat-induction factor of these peptides in the Δ*ctsR* mutant than in the wild type strain, due to a lower basal level in the mutant grown at 30 °C (Table 1). Overall, there was a moderate increase of the general stress-induced chaperones GroES, GroEL and DnaK (spots 6, 7 and 8) ranging from 1.25-fold to 5.7-fold upon temperature upshift in both strains analyzed (Figure 1). The levels of other two peptides Hsp1 (spot 2), Hsp3 (spots 3–5), drastically increased when bacteria were submitted to heat-stress at 42 °C. In particular, the small heat shock protein Hsp1 (spot 2) was never detected in extracts from cultures grown under optimal temperature conditions. Similarly, spot 4 was absent in the mutant and only weakly detectable in the *L. plantarum* WCFS1 wild type untreated cultures (Figures 1 and 2). Spots 3, 4 and 5 were identified as the same heat shock protein, Hsp3, thus revealing the existence of three potential isoforms, exhibiting two different molecular mass (Mr) (about 16.9 kDa for spot 4 and 15.4 kDa for spots 3 and 5) and pI values (approximately pH 5.0 for spot 5 and pH 4.8 for spots 3 and 4) (Figure 2). Spot 5 showed the highest heat-induction, increasing up to approximately 30-fold in both strains, and exhibited a Mr 1.2 kDa lower than the predicted theoretical mass, thus suggesting a potential post-translational modification connected to heat shock response. Proteomics should serve as a highly reproducible tool to allow the identification of spots by comparing maps obtained from different studies. Indeed, we could recognize the characteristic three spots of Hsp3 even in previously reported 2D-gel analyses of total extracts from different *L. plantarum* strains [18,20]. Izquierdo and coauthors [20] detected only one spot; while, in contrast to our results, Hamon *et al.* [18], identified two of these three spots as the alkaline shock proteins Asp1 and Asp2. Therefore, to our knowledge, this would be the first study to indicate the existence of different isoforms of small heat shock proteins in *L. plantarum* WCFS1 as well as in other LAB species. Future studies should address this aspect especially in relation to possible mechanisms of regulation of sHsp activity.

### 2.3. Influence of Δ*ctsR* Mutation in Protein Expression Levels

Two spots (9 and 10) clearly differentiated between the mutant and the wild type proteomic map under all experimental conditions. Spot 9 was only present in the *L. plantarum* WCFS1 wild type protein extracts (Figure 1A,C), whereas spot 10 was only observed in the mutant strain samples (Figure 1B,D). These spots, slightly differing only in the isoelectric point value (5.13 and 5.17, respectively), were both identified as the seryl-tRNA synthetase SerRS2 (Figure 2). Intriguingly, the *serRS2* gene of *L. plantarum* WCFS1 is located exactly upstream of *ctsR*. Even if the correct *ctsR* deletion had been previously verified [13], we cannot rule out the occurrence of polar effects that may have altered the expression of proximal genomic regions, including *serRS2*. Maybe such alteration could be associated to the detected post-translational modification. Aminoacyl-tRNA synthetases are essential components in the translation machinery in all forms of life. They are responsible for charging their cognate tRNAs with the correct amino acid [21]. We do not know the biological significance of this observed difference. To our knowledge, no previous proteomic study has reported similar results. We can also hypothesize that the form found in the Δ*ctsR* extracts might arise from pleiotropic effects of the mutation.

A set of 13 spots (11–23, Figures 1 and 2 and Table 1) showed a differential pattern in the two strains analyzed. Under optimal growth temperature, the levels of these peptides were higher (from 1.75- to 12-fold) in the mutant. After heat shock, the spots increased in intensity in both strains, the resulting peptide levels being on average 1.8-fold higher in the Δ*ctsR* mutant than in its parental strain. MASCOT analysis revealed that these spots were isoforms of just three proteins, namely ClpP, ClpE and ClpB. Summing together the spots recognized as the same protein showed that the levels of all of them were enhanced as a consequence of the Δ*ctsR* mutation without the need of heat-stress. Thus, ClpB, ClpP and ClpE levels were, respectively, approximately 11-, 6- and 5-fold higher in the Δ*ctsR* mutant than in the parental strain (Table 1). This finding is consistent with previous transcriptional analysis [14], and further confirms that *clp* genes belong to the CtsR regulon of *L. plantarum* WCFS1. Indeed, potential CtsR boxes were previously found in their 5′ regions. Moreover, in line with the proteomic data presented here, their basal mRNA level was found to be higher in the Δ*ctsR* deficient strain, indicating lack of repression by CtsR [14]. Analyzing *L. plantarum* cultures exposed to alkaline conditions, Lee *et al.* [19] observed that an increase in ClpB level corresponded to a reduction in the DnaK concentration. Similarly, in this study, we found that typical stress-response proteins, such as DnaK, GroEL, GroES, the small heat shock proteins Hsp1 and Hsp3 and the cold shock protein CspC were always downregulated in the unstressed mutant strain. Our interpretation is that the notably higher levels of proteins belonging to the Clp family observed in the Δ*ctsR* mutant could provide the correct refolding or proteolytic degradation of the damaged proteins at physiological conditions, thus limiting the activation of other chaperone systems.

**Figure 3:**
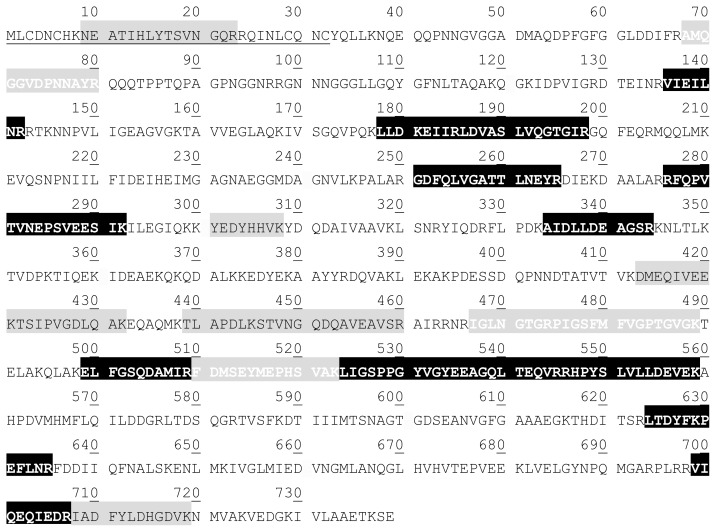
Amino acid sequence of *L. plantarum* WCFS1 ClpE. The *N*-terminal zinc finger region is underlined. The peptides identified only after trypsin digestion of the four ClpE isoforms with heavier Mr in black font on grey background, those identified from trypsin digestion of the three lighter Mr isoforms are in white font on grey background. The peptides found after trypsin digestion of all isoforms are on black background.

We detected two putative isoforms (spots 12 and 13) of the 21.5 kDa proteolytic subunit ClpP. Spot 11 was also identified as ClpP, although its molecular mass (33 kDa) and isoelectric point did not correspond to the theoretically predicted ones. This particular form of ClpP was previously observed by Cohen *et al.* [15] in *L. plantarum* WCFS1 at stationary-phase, suggesting that this might be due to a complex with an unidentified small molecule. Three isoforms (spots 21–23) with a Mr of 95.6 kDa were identified as the ATP-binding subunit ClpB, varying slightly in their isoelectric points (Figure 2). Seven well-defined spots (14–20) were identified as being isoforms of the ATP-binding subunit ClpE. These peptides were resolved in the gel into two main groups (four, spots 17–20, and three, spots 14–16) with a Mr difference of 5.8 kDa (Figure 2). Recently, Elsholz *et al.* [7] demonstrated that the heat stress regulation of CtsR was regarded to be dependent on an intrinsic heat-sensing property of CtsR in all low GC, Gram-positive bacteria. Therefore, de-repression is considered as a physiological process, which is independent from CtsR degradation. It was reported that ClpE plays a role in the control of CtsR stability, but the mechanisms of how this occurs has not yet been clarified [7,22–24]. Two ClpE forms, differing by 6 kDa in their Mr, as revealed by western blot analysis, were previously reported in extracts from *Lactococcus lactis* strains subjected to either high temperature [22] or oxidative stress [24]. Based on these previous studies and on our data, we can therefore suggest that a variation of the relative concentration of these forms may be associated to a cellular heat stress condition. However, in contrast to what was observed by Elsholz *et al.* [24], and taking into account our proteomic analyses, it is the small subunit that seems to characterize physiological conditions in the wild type, whereas the heavier Mr isoforms appear only in response to heat shock. Accordingly, in the mutant strain, which constitutively exhibits a proteomic pattern that is typical of a stress condition, all ClpE forms are evident in extracts from both unstressed and heat stressed cells. The peptide mass fingerprint of the four heavier isoforms revealed the occurrence of a 15 amino acid fragment corresponding to the *N*-terminal zinc finger region (Figure 3). However, this peptide was never detected in the digestion pattern of the three small ClpE isoforms; instead, a different 13 amino acids *N*-terminus proximal fragment, starting from position 66, was found. Such a fragment was never observed after trypsin digestion of ClpE spots with heavier Mr (Figure 3).

This finding may suggest a putative cleavage site after the N-terminal zinc finger region that justifies the observed difference of 5.8 kDa between the two ClpE groups, although, to confirm it, *N*-terminus of the different spots should be experimentally determined. The zinc finger motif in the *N*-terminal domain is highly conserved in the ClpE family [25]. Varmanen *et al.* [22] reported that in heat stressed *L. lactis*, the small ClpE isoform was lacking in a Δ*clpP* mutant, while the same isoform prevailed in bacteria expressing a mutation in the ClpE zinc finger domain, suggesting that this region is implicated in ClpP-dependent processing. Recently, Miethke and coauthors [23] suggested that ClpEP is involved in CtsR degradation after heat shock and demonstrated that the ClpE *N*-terminal zinc finger domain is essential for basal *in vitro* ATPase activity. Elsholz *et al.* [7] found that, during heat exposure, CtsR concentration diminished in *L. lactis* wild type but it was not affected in a Δ*clpE* mutant, confirming that a ClpEP complex is responsible for CtsR quality control in low GC, Gram-positive bacteria lacking McsA and McsB modulators. Strikingly, we found that, in unstressed wild type strain, the small ClpE variant was 10-fold more abundant than the higher molecular mass ClpE. Conversely, in the mutant strain and when wild type cells were exposed to heat, the ratio between both forms was unchanged (almost 1:1 ratio). According to the reported studies, our results suggest that when *L. plantarum* WCFS1 cells are exposed to heat stress, a ClpEP complex may bind CtsR via a putative ClpE zinc finger domain interaction, allowing CtsR degradation and, thus, the expression of the CtsR regulon.

### 2.4. Transcriptional Analysis of Relevant Genes

**Figure 4:**
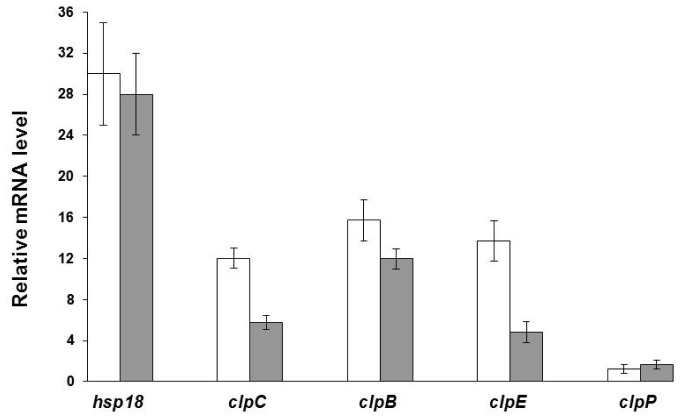
Transcriptional heat induction of putative CtsR regulon genes. Heat induction of putative CtsR regulon genes in *L. plantarum* WCFS1 wild type and Δ*ctsR* mutant strains as revealed by qRT-PCR analysis. The mRNA levels in wild type (open bars) and Δ*ctsR* mutant (grey bars) strains were calculated relative to the corresponding unstressed control cultures. RNA was extracted and analyzed from mid-exponentially growing cultures before (control) and after 10 min temperature up-shift to 42 °C (heat stress). *ldhD* was used as internal control gene. Data shown are mean ± SD of three independent experiments. For all genes except *clpP*, heat induction values were statistically significant with respect to control conditions (*p* ≤ 0.05).

Changes in the expression of the genes encoding the proteins under investigation and/or under putative CtsR control were monitored by qRT-PCR transcriptional analysis. The mRNA levels in both wild type and mutant strains were analyzed for *clpE*, *clpB*, *clpP* and *hsp1*, identified in the protein map, as well as for the *clpC* gene, whose promoter, like that of *hsp1*, was previously shown to be specifically recognized and bound by the recombinant *L. plantarum* WCFS1 CtsR [14]. In order to evaluate heat stress induction at the transcriptional level, the mRNA pattern of these *L. plantarum* WCFS1 genes, presenting CtsR operators, was monitored prior to and after short heat exposure, in both wild type and mutant strains. Relative to control temperature condition, increased mRNA levels were detected after heat shock for all the analyzed genes in both strains (Figure 4). Overall, the transcriptional profile matched the proteomic data (stress induction factors of Table 1), except for *clpC*, whose protein could not be identified by the 2D-gel analysis, and *clpP* level in the wild type, whose mRNA was poorly heat-induced (1.3 fold), in contrast to the increased protein concentration (4.9 fold).

In the parental strain, *hsp1*, *clpC*, *clpB*, and *clpE* transcripts were strongly induced by heat shock, confirming proteomic analysis and their involvement in heat-stress response mechanisms. In contrast, *clpP* expression was poorly heat induced (1.7-fold) in the mutant strain and only weakly triggered in the wild type (1.3-fold); such a discrepancy might reflect a different kinetics/temporal pattern of transcriptional activation [26] and/or a particularly short half-life of the *clpP* mRNA. Indeed, the transcriptional induction of some CtsR-controlled genes, including *clpP*, was found to be rather transient in response to heat shock [7,22,27].

The significant heat induction of putative CtsR regulon genes, at both transcriptional and protein level, in the Δ*ctsR* mutant, suggests that additional, CtsR-independent, regulatory mechanisms contribute to control their expression. A heat shock control mediated by additional regulators was previously hypothesized in *L. plantarum* WCFS1 for the CtsR-controlled *ftsH*, as well as in other bacterial species for other CtsR-repressed genes [5,13,28–30]. The heat shock induction was particularly marked for *hsp1* (28-fold increase in the mutant strain), which is likely to be under a dual repression control, mediated by both CtsR and HrcA, as suggested by previous studies revealing the presence of a CIRCE element in *hsp1* promoter [9,31]. This hypothesis could also explain the failure in detecting Hsp1 in the proteomic map of both wild type and CtsR-deficient strains at optimal growth temperature. Possibly, under physiologic conditions, the strong repression exerted by CtsR and HrcA keeps the Hsp1 level below the detection limit for 2D-gel analysis.

For *clpC*, *clpB* and *clpE*, a lower transcriptional heat-induction was detected in the Δ*ctsR* genetic background, compared to wild type (Figure 4). These results correlate with the proteomic data on ClpB and ClpE: under physiological conditions, their basal level in the Δ*ctsR* strain was found to be higher than in the wild type; accordingly, giving the lack of repression by CtsR, a lower heat induction was expected and indeed observed in response to heat stress (Table 1). A similar pattern was observed for *clpC*, whose changes in expression could only be appreciated at the mRNA level, since the corresponding protein was not detected in the proteomic map of either wild type or Δ*ctsR L. plantarum*. We suppose that ClpC level may be below the detection limit in both strains, either in normal or heat stress condition. As a support to our hypothesis, other proteomic studies, using similar 2-D gel techniques, did not detect ClpC in extracts from *L. plantarum* cultures either under control conditions or subject to different kinds of stress [15–20].

Under optimal temperature conditions, the concentration of CspC (spot 1) was 37% lower in the mutant relative to wild type strain (Table 1). A lower expression of *cspC* gene in the Δ*ctsR* strain was also confirmed by qRT-PCR analysis, indicating a 50% decrease in its mRNA level with respect to wild type, as detected under physiologic conditions (data not shown). Sequence analysis of the *cspC* five prime untranslated region (5′-UTR) revealed the presence of a putative CtsR-binding site (5′-AGGTAAT TTT GGTCAGA-3′), which partially matches the consensus CtsR box (5′-RGTCADN NAN RGTCADN-3′), defined so far for Gram-positive bacteria [5]. However, based on our data, a direct CtsR repression on transcription of this gene appears unlikely.

## 3. Experimental Section

### 3.1. Bacterial Strains and Experimental Growth Conditions

The reference strain *L. plantarum* WCFS1 [32] and the previously described *L. plantarum* Δ*ctsR* deletion mutant [13] were used in this study. Strains were routinely grown in MRS broth (Pronadisa, Madrid, Spain) at 30 °C.

### 3.2. Preparation of Protein Extracts

Total protein extracts of *L. plantarum* strains were obtained from exponentially growing cultures (OD_600_ = 0.6) before (control) or after transfer from 30 °C to 42 °C and further incubation for 30 min (heat stress). Growth curve analyses were performed to assure that both strains were stressed at corresponding growth phases. Colony forming units (CFU) counting indicated similar survival rates at heat challenge for both strains. Cells were sedimented by centrifugation (11,000× *g*, 20 min, 4 °C), washed with PBS at pH 7.1, and pellets frozen at −70 °C. Total protein extracts were obtained by mechanical disruption with a French Press as previously described by Mohedano *et al.* [33]. The protein concentration in the extracts was quantified by using the Quant-iT™ Protein Assay Kit (Invitrogen, Carlsbad, CA, USA) according to the manufacturer’s protocol. All experiments were performed in triplicate.

### 3.3. Two-Dimensional (2D) Gel Analysis and Image Acquisition

Samples of the bacterial lysates, containing 80 μg of proteins, were analyzed by 2D electrophoresis as previously described [34] with the following modifications. The first dimension was run on IPG strips (pH 4.0–7.0, 17 cm) (Bio-Rad, Hercules, CA, USA) with a 4-step program (50 V for 16 h, 300 V for 45 min, 3500 V for 22 h 45 min, and 5000 V for 30 min). A total of >80,000 V h were reached in all cases. The second dimension was run on 12% SDS-PAGE at 5 watts/gel for 30 min and then at 17 watts/gel until the die-front reached the bottom edge (approximately 5 h). Reference markers consisted of 10 μL of Precision Plus ProteinTM Standards Dual Color (Bio-Rad).

The gels were stained with SYPRO Ruby protein gel stain (Bio-Rad) according to the manufacturer’s instructions, and images of the gels were taken with a EXQuest Spot Cutter (Bio-Rad).

### 3.4. 2D-Gel Image Analysis

The spots were detected and quantified with the PDQuest 2D analysis 7.4.1 software (Bio-Rad). For this quantification, 305 stained spots were matched in the 12 gels analyzed (three biological replicates for each experimental condition), and used to normalize the average intensity. In order to define the appropriate spot detection parameters, the faintest, the smallest and the largest spots were marked on the gel scan, followed by manual matching of landmarks and significant spots. Intensities were measured via both absolute and normalized spot volumes. Differentially expressed proteins were highlighted by statistical analysis. Spots showing statistically significant differences (Student’s *t* test, *p*-value ≤ 0.05) and a minimum of a 1.8-fold change of intensity were selected for identification. When different spots were identified as being the same protein (see 2.1), the total volume of the various isoforms was calculated.

### 3.5. Identification of Proteins by MALDI-TOF-TOF Peptide Mass Fingerprinting

Spots of interest were automatically excised using the EXQuest Spot Cutter, deposited in 96-well plates and analyzed at the Centro de Investigaciones Biológicas Proteomic Unit as follows. The spots were digested in gel automatically using a DigestPro MS apparatus (Intavis AG, Koeln, Germany). The digestion protocol used was based on Schevchenko *et al.* [35] with minor variations: gel pieces were washed first with 50 mM ammonium bicarbonate (Sigma-Aldrich, St. Louis, MO, USA) and second with acetonitrile (ACN) (Scharlau, Barcelona, Spain). Trypsin (Promega, Madison, WI, USA), at a final concentration of 12.5 ng/μL in 50 mM ammonium bicarbonate solution, was added to the gel pieces for 8 h at 37 °C. Finally, 70% ACN containing 0.5% trifluoroacetic acid (TFA) (Sigma-Aldrich) was added for peptide extraction. The extracted peptides were dried by speed-vacuum centrifugation and resuspended in 4 μL of 30% ACN with 0.1% TFA.

One microliter of each peptide mixture was deposited onto an 800 μm AnchorChip (Bruker- Daltonics, Bremen, Germany) and dried at RT. One microliter of matrix solution (3 mg/mL α-cyano-4-hydroxycinnamic acid) in 33% ACN with 0.1% TFA was then deposited onto the digest and allowed to dry at room temperature (RT).

Samples were analyzed with an Autoflex III TOF/TOF mass spectrometer (Bruker-Daltonics). Typically, 1000 scans for peptide mass fingerprinting (PMF) and 2000 scans for MS/MS were collected. Automated analysis of mass data was performed using FlexAnalysis software (Bruker-Daltonics). Internal calibration of MALDI-TOF mass spectra was performed using two trypsin autolysis ions with *m*/*z* 842.510 and *m*/*z* 2211.105; for MALDI-MS/MS, calibrations were performed with fragment ion spectra obtained for the proton adducts of a peptide mixture covering the *m*/*z* 800–3200 region. The typical error observed in mass accuracy for calibration was usually below 20 ppm. MALDI-MS and MS/MS data were combined through the BioTools 3.0 program (Bruker-Daltonics) to interrogate the NCBI non-redundant protein database (NCBI: 20100306) using MASCOT software 2.3 (Matrix Science, London, UK). Relevant search parameters were set as follows: enzyme, trypsin; fixed modifications, carbamidomethyl (C); oxidation (M); 1 missed cleavage allowed; peptide tolerance, 50 ppm; MS/MS tolerance, 0.5 Da. Protein scores greater than 75 were considered significant (*p*-value ≤ 0.05).

### 3.6. Quantitative RT-PCR Analysis

Total RNAs were extracted from exponentially growing *L. plantarum* cultures (OD_600_ = 0.6), before and after heat stress (incubation at 42 °C for 10 min) using the UltraClean microbial isolation kit (MO BIO, Carlsbad, CA, USA), according to the manufacturer’s instructions. The quality of the RNA samples, estimated by integrity of rRNA, was verified by electrophoresis on 1.2% agarose gels and analysis with Quantity One software (Bio-Rad). Total RNA preparations were quantified by using the Quant-iT™ RNA Assay Kit (Invitrogen, Carlsbad, CA, USA) according to the manufacturer’s protocol. One microgram of total RNA was reverse transcribed to cDNA using the Quantitect reverse transcription kit (Qiagen, Valencia, CA, USA), which includes a DNase I treatment step. The absence of chromosomal DNA contamination was confirmed by real-time PCR on corresponding DNase I-treated RNAs.

Quantitative real-time PCR (qRT-PCR) was performed on an Applied Biosystems 7300 real-time PCR instrument using SYBR green I detection. The *ldhD* gene of *L. plantarum* WCFS1 was used as internal control [14]. Each reaction mixture contained 5 μL of 20-fold-diluted cDNA, 10 μL of Power SYBR green PCR master mix (Applied Biosystems, Foster City, CA) and 100 nM of each sense and antisense primer [14]. The *cspC* forward and reverse primer sequences used were: 5′ ATC ACT CGC GAA AAC GGT AG 3′; and 5′ CGA TCG CTT TCT TCA ACG TC 3′, respectively. The amplification profile comprised 10 min at 95 °C, followed by 35 cycles consisting of 20 s at 95 °C, 30 s at 58 °C, and 30 s at 72 °C. Fluorescence was monitored during each extension phase, and melting-curve analyses were performed to confirm the amplification of specific transcripts. Data were analyzed using the AB 7300 software, by applying the two standard-curves quantification method. Each assay included triplicates of PCR of the samples, negative no-template controls, and standard curves for both the internal-control and target genes. Using one-tailed, two-tailed Student’s *t*-test, any *p*-value ≤ 0.05 was considered significant.

## 4. Conclusions

In this study, a comparative proteomic analysis was performed using a previously genotypically characterized Δ*ctsR* mutant of *L. plantarum* WCFS1. Comparisons between physiologic and heat stress conditions confirm the induction of typical stress response proteins, including different isoforms of Hsp3. Major differences between parental and Δ*ctsR* strains include the variation of the SerS2 pI and an apparent induction of ClpB, ClpP and ClpE, thus confirming their transcriptional regulation by CtsR. In this regard, *hsp1* could be the first case of *hsp* genes to be under a dual negative control mediated by both CtsR and HrcA in LAB. Moreover, the proteomic approach performed in this study reveals the presence of potential ClpE isoforms with two different Mr. The relationship between ClpE isoforms and CtsR may be a worthwhile goal in order to improve our knowledge in CtsR control in lactic acid bacteria.

## Figures and Tables

**Table 1:** Characteristics of the peptides with altered levels in control and heat-stressed cultures of *L. plantarum* WCFS1 wild type and Δ*ctsR* mutant strains. The induction factors are reported, considering both the differential expression between Δ*ctsR* and wild type strains (Lp Δ*ctsR*/Lp WCFS1) under control and heat stress conditions, and the relative induction within each strain upon heat stress. When a spot was not detected in the gel, we use the term On/Off for the calculation of ratio.

SSP	Identified Protein	Mascot Score	Sequence Coverage (%)	Peptides (*n*)	MW (kDa)/pI		Induction Factor			

					Theoretical	Observed	Lp Δ*ctsR*/Lp WCFS1		Stress/Control	

							Control	Stress	Lp WCFS1	Lp Δ*ctsR*
1	cold shock protein CspC	122	68	3	7.30/4.57	11.21/4.40	0.63 ± 0.06	1.15 ± 0.32	0.64 ± 0.05	1.16 ± 0.33
2	small heat shock protein Hsp1	340	54	8	15.99/4.53	14.32/4.50	n.d.	1.11 ± 0.24	On/Off	On/Off
3	small heat shock protein Hsp3	224	51	6	16.66/5.00	15.33/4.79	0.56 ± 0.09	1.56 ± 0.18	4.49 ± 1.09	9.36 ± 1.10
4	small heat shock protein Hsp3	255	51	6	16.66/5.00	16.93/4.76	Off/On	1.72 ± 0.14	12.72 ± 3.68	On/Off
5	small heat shock protein Hsp3	212	51	5	16.66/5.00	15.45/4.99	0.72 ± 0.44	0.83 ± 0.11	27.23 ± 1.21	31.29 ± 4.31
	∑ small heat shock protein Hsp3						0.75 ± 0.25	1.02 ± 0.12	14.85 ± 1.38	19.97 ± 2.35

6	GroES co-chaperonin	291	50	4	10.28/4.95	11.39/5.02	0.64 ± 0.09	0.90 ± 0.14	4.03 ± 0.26	5.70 ± 0.89
7	GroEL chaperonin	319	22	9	57.40/4.69	56.60/4.67	0.68 ± 0.10	0.96 ± 0.15	2.47 ± 0.17	3.51 ± 0.53
8	molecular chaperone DnaK	481	41	14	66.69/4.68	67.33/4.67	0.62 ± 0.07	0.94 ± 0.14	1.25 ± 0.06	1.87 ± 0.28
9	Seryl-tRNA synthetase SerRS2	282	13	4	48.22/5.16	48.41/5.13	Off/On	Off/On	0.69 ± 0.20	n.d.
10	Seryl-tRNA synthetase SerRS2	327	19	6	48.22/5.16	48.41/5.17	On/Off	On/Off	n.d.	0.67 ± 0.49
11	ATP-dependent Clp protease proteolytic subunit ClpP	129	42	5	21.51/4.87	32.90/5.24	1.75 ± 0.19	1.14 ± 0.20	3.20 ± 0.39	2.10 ± 0.36
12	ATP-dependent Clp protease proteolytic subunit ClpP	277	55	8	21.51/4.87	21.46/4.76	4.77 ± 0.68	1.99 ± 0.39	3.90 ± 0.60	1.63 ± 0.32
13	ATP-dependent Clp protease proteolytic subunit ClpP	154	37	5	21.51/4.87	21.46/4.88	On/Off	1.89 ± 0.10	On/Off	1.75 ± 0.09
	* ∑ ATP-dependent Clp protease proteolytic subunit ClpP						5.85 ± 0.84	1.97 ± 0.30	4.91 ± 0.60	1.65 ± 0.25

14	ATP-dependent Clp protease, ATP-binding subunit ClpE	492	27	14	81.90/5.28	77.09/5.15	3.41 ± 0.14	1.79 ± 0.17	2.88 ± 0.20	1.52 ± 0.14
15	ATP-dependent Clp protease, ATP-binding subunit ClpE	135	15	10	81.90/5.28	77.09/5.19	2.70 ± 0.13	1.49 ± 0.13	2.12 ± 0.11	1.17 ± 0.10
16	ATP-dependent Clp protease, ATP-binding subunit ClpE	529	20	10	81.90/5.28	77.09/5.22	4.12 ± 0.89	1.99 ± 0.28	2.52 ± 0.83	1.22 ± 0.17
17	ATP-dependent Clp protease, ATP-binding subunit ClpE	344	24	14	81.90/5.28	82.95/5.17	7.34 ± 2.26	2.15 ± 0.68	5.26 ± 0.86	1.54 ± 0.49
18	ATP-dependent Clp protease, ATP-binding subunit ClpE	450	23	13	81.90/5.28	82.95/5.20	12.08 ± 0.63	2.24 ± 0.03	8.66 ± 0.82	1.60 ± 0.02
19	ATP-dependent Clp protease, ATP-binding subunit ClpE	253	15	8	81.90/5.28	82.95/5.24	9.74 ± 1.20	1.68 ± 0.40	9.39 ± 1.03	1.62 ± 0.39
20	ATP-dependent Clp protease, ATP-binding subunit ClpE	509	20	10	81.90/5.28	82.95/5.28	On/Off	2.32 ± 0.63	On/Off	1.17 ± 0.32
	∑ ATP-dependent Clp protease, ATP-binding subunit ClpE						4.64 ± 0.45	1.66 ± 0.25	3.50 ± 0.25	1.26 ± 0.19

21	ATP-dependent Clp protease, ATP-binding subunit ClpB	279	25	17	96.51/5.19	95.63/5.12	On/Off	2.10 ± 0.08	On/Off	3.82 ± 0.14
22	ATP-dependent Clp protease, ATP-binding subunit ClpB	228	25	16	96.51/5.19	95.63/5.16	7.12 ± 1.42	1.82 ± 0.19	12.13 ± 4.92	3.10 ± 0.32
23	ATP-dependent Clp protease, ATP-binding subunit ClpB	378	35	22	96.51/5.19	95.63/5.19	On/Off	1.83 ± 0.22	On/Off	2.86 ± 0.35
	∑ ATP-dependent Clp protease, ATP-binding subunit ClpB						10.74 ± 2.19	1.83 ± 0.29	17.23 ± 5.77	2.93 ± 0.77

n.d.: spot not detected in both conditions compared.

* ∑ ATP-dependent Clp protease proteolytic subunit ClpP did not take into consideration spot 11.
